# The moderating role of race/ethnicity and nativity in the relationship between perceived discrimination and overweight and obesity: results from the National Epidemiologic Survey on Alcohol and Related Conditions

**DOI:** 10.1186/s12889-019-7811-0

**Published:** 2019-11-06

**Authors:** Adolfo G. Cuevas, Kasim Ortiz, Yusuf Ransome

**Affiliations:** 1 Department of Community Health, Tufts University, 574 Boston Ave, Suite 208, Medford, MA 02155 USA; 2 Department of Sociology & Criminology, University of New Mexico, MSC05 3080, 1915 Roma NE Ste. 1103, Albuquerque, NM 8713 USA; 3 Department of Social & Behavioral Sciences, Yale School of Public Health, 60 College Street, LEPH Building, New Haven, CT 06510 USA

**Keywords:** Race/ethnicity, Discrimination, Nativity, Obesity

## Abstract

**Background:**

The overweight/obesity epidemic is a public health issue in the United States (US), that disproportionately affect certain racial/ethnic minority groups. Perceived discrimination has been implicated as a health risk factor. However, research on race/ethnicity, perceived discrimination, and obesity has been mixed. Researchers suggest that perceptions of discrimination may be dependent upon nativity status. This study evaluated the role that nativity status and race/ethnicity play in the relationship between perceived discrimination and overweight/obesity.

**Methods:**

We used Wave 2 of the National Epidemiologic Survey on Alcohol and Related Conditions (2004–2005) [*N* = 33,319]). Multinomial logistic regression assessed a three-way interaction (perceived discrimination × race/ethnicity × nativity) on overweight and obesity, adjusting for sociodemographic factors and health-related behaviors.

**Results:**

The three-way interaction was significant for overweight [F (17, 49) = 3.35; *p* < 0.001] and obesity [F (17, 49) = 5.05; *p* < 0.001]. Among US-born individuals, US-born non-Hispanic Blacks had a decreased risk of being obese compared to US-born non-Hispanic Whites at mean levels of perceived discrimination [aRRR = 0.71; 95% CI (0.51–0.98); *p* = 0.04). Among foreign-born individuals, foreign-born South Americans had an increased risk of being overweight at mean levels of perceived discrimination compared to foreign-born non-Hispanic Whites [aRRR = 8.07; 95% CI (1.68–38.77); *p* = 0.01], whereas foreign-born Dominicans had a decreased risk of being obese compared to foreign-born non-Hispanic Whites [aRRR = 0.05; 95% CI (0.01–0.20); *p* < 0.001].

**Conclusion:**

Perceived racial discrimination is a risk factor for overweight/obesity for certain groups. Race/ethnicity and nativity may play important roles in the relationship between perceived discrimination and overweight/obesity. Future research is needed to identify the behavioral and psychological pathways that link perceived discrimination and overweight/obesity.

## Introduction

The overweight/obesity epidemic has been a persistent public health issue in the US, disproportionately affecting certain racial/ethnic minority groups [1, 2]. Non-Hispanic Blacks and Hispanics/Latinos have higher prevalence of overweight/obesity compared to non-Hispanic Whites [2], which places them at a greater risk for obesity-related diseases, such as hypertension, coronary heart disease, and stroke [1, 3–6]. There is also evidence that individuals who are overweight are at increased risk of cardiovascular disease risk [7] and are more likely to become obese over time [8]. Nevertheless, these aggregate data mask important variations in overweight/obesity based on nativity status. Limited evidence shows that US-born individuals, across race/ethnicity, have significantly higher obesity prevalence compared to their foreign-born counterparts [9, 10]. For instance, US-born Blacks are approximately 2.5 times more likely to be obese than foreign-born Blacks, whereas US-born Whites and US-born Hispanics are each 1.4 times more likely to be obese compared to their foreign-born counterparts [10].

The etiology of obesity and obesity disparities is multifactorial, reflecting a complex interaction of biobehavioral and socioenvironmental factors [7]. For instance, there is evidence that genetic markers, such as a variant in *FTO* (rs9939609) independently increases obesity susceptibility [7]. Other factors, such as physical inactivity and poor dietary behaviors, interact together to further increase risk of obesity [7, 11, 12]. Racial/ethnic minorities generally engage in less physical activity and consume fewer fruits and vegetables than Whites [13]. These obesity-related behaviors are thought to contribute to racial/ethnic disparities in obesity [14]. Given that racial/ethnic minorities are disproportionately at the lower end of the socioeconomic strata as compared to Whites, researchers have suggested racial/ethnic differences in obesity may simply be a function of underlying differences in socioeconomic status, via differences in social environments that facilitate healthy dietary and exercise behaviors [15–17].

Studies have found, however, that the association between SES and obesity varies by race/ethnicity and that ethnic/racial differences in obesity are not fully explained by SES [15, 18–20]. Moreover, despite foreign-born individuals disproportionately being at the lower end of the SES strata, they display better health, lower mortality, and lower obesity rates than their US-born counterparts who tend to have higher levels of income and education [10, 21–25]. While US-born individuals engage in different health behaviors (e.g., dietary intake and physical activity) compared with foreign-born, even after adjusting for health behavior and SES, differences in nativity status persist [10].

Stress can increase the risk of overweight and obesity through psychological means [26, 27]. For example, greater reports of stressful life events are associated with increased reports of depressive symptoms [28]. There is compelling evidence that depression increases the risk of obesity [29]. Stress can also increase the risk of overweight and obesity through behavioral pathways. Animal and human studies show that stress can induce cravings for high sugar and high fat foods [30]. Moreover, stress can reduce effort to engage in physical activity [31].

Perceived discrimination operate like other stressors, in that they are life-long and cumulative and can lead to illness and disease [32–34]. Growing attention has been given to the ways in which race/ethnicity-related aspects of social experiences, particularly perceived discrimination, may increase the risk of obesity [35–37]. Findings pertaining to the relationship between discrimination and obesity, however, have been mixed. For example, Molina and colleagues found that among 602 Latino adults living in Lawrence, MA, those who reported greater general perceived discrimination were more likely to have higher BMI and waist circumference, even after adjusting for sociodemographic factors, physical activity, and stressful life events. In a multi-racial/ethnic sample of 3105 adults, greater reports of discrimination (both racial and non-racial) were associated with increased abdominal obesity, but only among ethnic Whites (e.g., Irish, Jewish, Polish) [37]. Lewis and colleagues found that, among White and Black women (*N* = 402; 45% African-American, 55% Caucasian), greater reports of general discrimination were associated with higher amounts of visceral fat, but the association did not vary by race [38]. Vines and colleagues found that, among 447 Black women, perceived racism was inversely associated with lower levels of waist-to-hip ratio [39].

As research continues to document the relationship between perceived discrimination and obesity, greater attention is needed on the heterogeneity within racial/ethnic groups. Brondolo and colleagues [40] suggest that perception of discrimination, particularly among racial/ethnic minority groups, may be dependent on an individual’s membership to other social identity groups. Nativity status is one group membership that can influence perception of discrimination. Because foreign-born individuals are typically born and raised in a society where their racial/ethnic group is the majority, they may have had fewer experiences of discrimination based on their race/ethnicity in their country of origin. Latino/Hispanic immigrants, in particular, are from countries with distinct social construction of race. Going back to colonialism, Latinos with phenotypes of African descent were the lowest in the social hierarchy, followed by those of indigenous descent; while those who were phenotypically European held political, social, and economic power [41]. Much of these inequalities persist and manifest in Latin American countries today in overt and subtle ways, with darker skin and African phenotypic features being perceived as less desirable, while lighter skin and European phenotypic features perceived more favorably [41]. This form of discrimination (i.e., colorism) may be more salient to Hispanic/Latino immigrants living in the US than discrimination based on their race/ethnicity. Moreover, Hispanic/Latino immigrants living in the US experience unique stressors, like acculturative stress (i.e., the psychological impact of adapting to a new culture) [42], which may be a more immediate threat to the self than racial/ethnic discrimination. US-born individuals, on the other hand, are exposed to more discussions and studies of race and racism, which can potentially make them more adept at recognizing racial/ethnic bias in the US [40, 43, 44]. For US-born racial/ethnic minorities, in particular, race/ethnicity may be a more salient source of individuals’ perceptions of discriminatory treatment than other social identities as they recognize that their social and economic conditions are shaped by institutional and interpersonal discrimination [45, 46].

In a study of 1454 urban-dwelling Asian and Black adults, U.S.-born individuals reported more race-related stigmatization and exclusion than foreign-born individuals. In a community-based study of 185 US-born and 114 foreign-born Black pregnant women, US-born Black pregnant women reported greater prevalence of personal racism and group racism compared to foreign-born Black pregnant women. Another study found that found greater reporting of discrimination (e.g., being treated with less courtesy than other people; being treated with less respect than other people) among US-born Latinos (47% vs. 25%) compared to foreign-born Latinos [47].

Perceived discrimination and its obesogenic sequelae are potentially obscured, without considering the intersection of race/ethnicity and nativity status. The effects of discrimination on overweight/obesity may be stronger for certain groups. A better understanding of the association between perceived discrimination and overweight status/obesity will allow us to identify those most vulnerable to discrimination. Using a multi-racial/ethnic, population-based sample of adults, we examined the interrelationship between racial/ethnic perceived discrimination, race/ethnicity, and nativity status on overweight/obesity. We hypothesize that the association between racial/ethnic perceived discrimination and overweight/obesity is stronger for US-born racial/ethnic minorities.

## Methods

We used data from Wave 2 of the National Epidemiologic Survey on Alcohol and Related Conditions (NESARC) [2004–2005] [48], which is a population-representative survey of United States adults living in noninstitutionalized settings. Trained interviewers utilized computer-assisted personal interviews to capture health outcomes, behavioral factors, and psychiatric disorders of respondents > 18 years of age [49]. NESARC data are weighted to adjust for the probabilities of selecting households, selecting one person per household, oversampling and nonresponse. There was also an oversampling of young adults (18–24 years old) and non-Hispanic Blacks and Hispanics/Latinos to ensure appropriate representation of racial and ethnic subgroups and to obtain reliable statistical estimation in these subpopulations [50]. Wave 2 involved re-interviews of 34,653 Wave 1 participants, resulting in a cumulative response rate of 70.2%. NESARC received ethical approval from the U.S. Census Bureau and the U.S. Office of Management and Budget [49]. The final sample for the current analyses included *N* = 32,747 respondents. This study was approved by the Institutional Review Board at Harvard T.H. Chan School of Public Health.

### Anthropometric measures

Trained interviewers administered survey-based measures and measured the respondent’s height and weight. The primary outcomes were overweight and obesity based on Body Mass Index (BMI). BMI was calculated based on measured height and weight, and values were categorized as follows: a) underweight/normal [BMI ≤ 25]; b) overweight [25.1 ≥ BMI ≤ 30]; and c) obese [BMI ≥ 30.1]. This coding follows previously utilized assessments of weight statuses using NESARC [51].

### Perceived racial discrimination

We measured perceived racial discrimination using the validated Experiences of Discrimination (EOD) instrument [52]. Respondents were asked how often in the past year they had “been prevented from doing something, or been hassled or made to feel inferior in any of the following situations because of your race, ethnicity, or color.” The EOD asks respondents whether they have experienced racial discrimination in nine different social context, including work, school, housing, and with the police and courts. Participants were given the option of 0 = Never, 1 = Once, 2 = Two or three times, or 3 = Four or more times.

### Race/ethnicity

We took full advantage of NESARC’s oversampling of racial minority populations and included Hispanic/Latino subgroups. Race/ethnicity was coded as follows: non-Hispanic White (reference), non-Hispanic Black, Mexican American, Cuban American, Puerto Rican, Central American, South American, Dominican, and non-Hispanic Other.

### Nativity

Nativity was assessed using the following question: “Were you born in the United States”, for which respondents were provided yes/no response option.

### Covariates

Due to their known relationship with either overweight/obesity or racial discrimination [53–56], we included the following socio-demographic covariates in the adjusted regression models: gender (male/female), educational attainment (less than high school, high school diploma/general education degree, some college/bachelor’s degree, graduate education), age (18–98 years of age); personal income (less than/equal to $19 K, $20 K- $35 K, $36 K - $70 K, more than $70 K), marital status [married/cohabitating, widowed/divorced/separated, never married), current smoking status (yes/no), and census region.

### Statistical analyses

First, we present sociodemographic characteristics of our study sample; wherein we conducted Rao-Scott *X*^2^ tests on categorical variables, comparing demographic markers across weight statuses. We then deployed multinomial logistic regressions to examine the three-way interaction (perceived discrimination × race/ethnicity × nativity) on overweight/obesity, adjusting for all covariates. Results of the regression models are reported using relative risk ratios (RRRs) with corresponding 95% confidence intervals (CI). All analyses were conducted using Stata 14.2 software, taking into consideration complex survey design of the NESARC, using Stata’s *svy: mlogit* suite of commands [57].

## Results

A total of 32,747 participants were included in the main analyses, of whom 9947 (28.34%) were obese, 11,329 (33.97%) were overweight, and 12,159 (37.68%) were normal or underweight (see Table 1). The average age of the sample was 45.08 years old. Less than half of the participants were male (47.92%). Non-Hispanic Whites had a greater proportion of normal weight/underweight than overweight and obese. For the other race/ethnicity groups, the weight distributions were relatively the same. Among men, the highest percentage of respondents were in the overweight group, whereas for women, the majority were found in the underweight/normal weight group. A greater proportion of non-smokers and smokers were normal weight/underweight than overweight and obese. Among US-born individuals, US-born non-Hispanic Blacks (M = 1.24; SE = 0.01) reported the highest level of discrimination compared to other US-born groups. Among foreign-born individuals, foreign-born Dominicans and non-Hispanic other had the reported the highest level of discrimination compared to other foreign-born groups (see Table 2).

**Table 1:** Weighted Descriptive Demographics by Weight Statuses: National Epidemiological Survey of Alcohol & Related Conditions (2003–2004); *N* = 32,747

	Normal/underweight %	Overweight %	Obese %	*p*-value
Race/Ethnicity				< 0.01
Non-Hispanic White	28.04%	24.17%	19.39%	
Non-Hispanic Black	3.17%	3.65%	4.47%	
Mexican	1.74%	2.49%	2.14%	
Cuban	0.16%	0.19%	0.13%	
Puerto Rican	0.37%	0.55%	0.39%	
Central American	0.44%	0.52%	0.31%	
South American	0.28%	0.29%	0.15%	
Dominican	0.10%	0.09%	0.07%	
Non-Hispanic Other	3.42%	1.97%	1.29%	
Marital Status				< 0.01
Married	22.57%	22.93%	18.31%	
Widowed/Divorce	7.49%	6.14%	5.36%	
Never Married/Single	7.62%	4.90%	4.67%	
Gender				0.06
Male	14.53%	19.49%	13.18%	
Female	23.16%	14.48%	15.16%	
Educational Attainment				< 0.01
Less than High School	4.68%	4.87%	4.57%	
High School Diploma/GED	9.27%	9.50%	8.82%	
Some College/College Graduate	17.77%	15.06%	12.25%	
Graduate School	5.97%	4.54%	2.70%	
Income				< 0.01
Less than $20,000	14.02%	9.95%	9.85%	
$20,000 – $35,999	11.71%	11.02%	9.10%	
$36,000 – $70,999	8.27%	8.83%	6.88%	
$71,000+	3.58%	4.18%	2.52%	
Current Smoking Status				< 0.01
No	28.60%	26.66%	22.69%	
Yes	9.03%	7.34%	5.68%	
Nativity				< 0.01
U.S. Born	31.91%	28.87%	25.32%	
Foreign-Born	5.77%	5.11%	3.03%	
Census Region				0.08
Northeast	6.70%	5.99%	5.06%	
Midwest	6.77%	6.34%	5.42%	
South	14.33%	13.00%	11.08%	
West	9.89%	8.64%	6.79%	
	M (SE)	M (SE)	M (SE)	
Discrimination (0–5)	1.08 (0.004)	1.08 (0.004)	1.09 (0.004)	< 0.01
Age	44.44 (0.250)	46.17 (0.234)	44.72 (0.225)	< 0.01

**Table 2:** Multinomial Logistic Regression: Racial Discrimination Levels, Results Stratified by Race/Ethnicity & Nativity: National Epidemiological Survey of Alcohol & Related Conditions – Wave 2 (2003–2004; *N* = 32,747)

Race/ethnicity	US-born	*P*-value
Non-Hispanic White	1.04 (0.002)	0.01
Non-Hispanic Black	1.24 (0.01)	
Mexican American	1.19 (0.02)	
Cuban	1.10 (0.03)	
Puerto Rican	1.19 (0.02)	
Central American	1.09 (0.03)	
South American	1.06 (0.02)	
Dominican	1.16 (0.05)	
Non-Hispanic Other	1.11 (0.02)	
	Foreign-born	
Non-Hispanic White	1.11 (0.01)	0.01
Non-Hispanic Black	1.17 (0.03)	
Mexican American	1.13 (0.01)	
Cuban	1.06 (0.02)	
Puerto Rican	1.14 (0.03)	
Central American	1.12 (0.02)	
South American	1.19 (0.04)	
Dominican	1.21 (0.10)	
Non-Hispanic Other	1.21 (0.02)	

### Main effects

In assessing the interaction between race/ethnicity and perceived discrimination, Dominicans [aRRR = 0.04; 95% CI (0.01–0.15); *p* = 0.001] and non-Hispanic Other respondents [aRRR = 0.50; 95% CI (0.30–0.84); *p* = 0.019) reporting greater perceived discrimination indicated a decreased relative risk of identifying as obese compared to their normal weight counterparts. When assessing the main effect of perceived discrimination and nativity, foreign-born respondents reporting greater perceived discrimination indicated a decreased relative risk of both overweight [aRRR = 0.64; 95% CI (0.51–0.82); *p* = 0.001] and obese [aRRR = 0.50; 95% CI (0.35–0.70); *p* = 0.001] compared to their U.S.-born counterparts. Table 3 provides estimates of interaction main effects of perceived discrimination and race/ethnicity and nativity respectively.

**Table 3:** Multinomial Logistic Regression Analysis of Weight Statuses Impacted by Racial Discrimination: National Epidemiological Survey of Alcohol & Related Conditions – Wave 2 (2003–2004; *N* = 32,747)

	Overweight vs. Normal/Underweight RRR 95% CI	Obese vs. Normal/Underweight RRR 95% CI
	Interactions	
Race/Ethnicity*Racial Discrimination		
Non-Hispanic White (ref)	1.00	1.00
Non-Hispanic Black	0.81 [0.60–1.11]	0.78 [0.56–1.08]
Mexican	0.92 [0.65–1.30]	0.65 [0.41–1.04]
Cuban	1.18 [0.42–3.29]	1.45 [0.61–3.43]
Puerto Rican	0.63 [0.31–1.28]	1.02 [0.51–2.03]
Central American	1.37 [0.56–3.36]	0.86 [0.37–2.02]
South American	2.38 [1.16–4.90]	1.20 [0.47–3.11]
Dominican	0.39 [0.11–1.41]	0.04*** [0.01–0.15]
Non-Hispanic Other	0.99 [0.62–1.57]	0.50** [0.30–0.84]
Nativity*Racial Discrimination		
U.S. Born (ref)	1.00	1.00
Foreign Born	0.64*** [0.51–0.82]	0.50*** [0.35–0.70]

All models are adjusted by age(y), personal income, marital status, smoking status, educational attainment, drinking status

^a^ RRR = Relative Risk Ratios

^b^ 95% CI = 95% Confidence Interval

*0.10; **0.050; ***0.001

### Three-way interaction

The three-way interaction was significant [F (32, 34) = 3.10; *p* < 0.001], as well as that for the overweight [F (17, 49) = 3.35; *p* < 0.001] and obese [F (17, 49) = 5.05; *p* < 0.001] categories respectively. We present nativity-stratified results of the interaction between perceived discrimination and race/ethnicity to compare and contrast the moderating effects of race/ethnicity and perceived discrimination on overweight/obesity (see Table 4). Results indicate that at mean levels of perceived discrimination, US-born non-Hispanic Blacks had a decreased risk of being obese compared to US-born non-Hispanic Whites [aRRR = 0.71; 95% CI (0.51–0.98); *p* = 0.04). Among foreign-born individuals, foreign-born South Americans had an increased risk of being overweight at mean levels of perceived discrimination [aRRR = 8.07; 95% CI (1.68–38.77); *p* = 0.01], whereas foreign-born Dominicans who reported greater perceived discrimination were at a decreased risk of being obese compared to foreign-born non-Hispanic Whites [aRRR = 0.05; 95% CI (0.01–0.20); *p* < 0.001].

**Table 4:** Multinomial Logistic Regression: Weight Statuses Impacted by Racial Discrimination, Results Stratified by Nativity: National Epidemiological Survey of Alcohol & Related Conditions – Wave 2 (2003–2004; *N* = 32,747)

	Overweight vs. Normal/Underweight RRR^a^ 95%CI^b^	Obese vs. Normal/Underweight RRR 95% CI	Overweight vs. Normal/Underweight RRR 95% CI	Obese vs. Normal/Underweight RRR 95% CI
	Nativity-Stratified Models			
	U.S. Born		Foreign-Born	
Race/ethnicity*Racial Discrimination				
Non-Hispanic White	1.00	1.00	1.00	1.00
Non-Hispanic Black	0.76 [0.54–1.07]	0.71** [0.51–0.98]	2.52 [0.57–11.13]	1.73 [0.64–4.69]
Mexican American	0.98 [0.63–1.56]	0.78 [0.48–1.27]	2.78 [0.65–11.92]	0.57 [0.16–2.07]
Cuban	2.06 [0.18–23.18]	2.67 [0.33–21.55]	3.94 [0.47–33.08]	1.52 [0.33–7.07]
Puerto Rican	0.72 [0.22–2.36]	1.66 [0.62–4.45]	2.22 [0.46–10.64]	0.76 [0.21–2.74]
Central American	3.16 [0.72–13.92]	0.63 [0.07–5.56]	2.99 [0.58–15.48]	1.19 [0.28–5.01]
South American	1.73 [0.15–20.26]	0.02 [0.00–4.14]	8.07* [1.68–38.77]	1.63 [0.33–7.93]
Dominican	4.11 [0.02 – N/A]	0.06 [0.00–38.50]	1.25 [0.24–6.64]	0.05*** [0.01–0.20]
Non-Hispanic Other	1.55 [0.81–2.97]	1.07 [0.56–2.03]	3.03 [0.61–14.96]	0.49 [0.13–1.81]

All models are adjusted by age(y), personal income, marital status, smoking status, educational attainment [Foreign-born models also adjust for length of time in the U.S.]

^a^ RRR = Relative Risk Ratios

^b^ 95%CI = 95% Confidence Interval

*0.10; **0.050; ***0.001

## Discussion

To the best of our knowledge, this was the first study to examine the interaction between perceived discrimination, race/ethnicity, nativity on overweight/obesity across a multiracial/ethnic sample of adults. We found a three-way interaction between perceived discrimination, race/ethnicity, and nativity status on obesity. Contrary to our expectations, we found that, at mean levels of perceived discrimination, US-born non-Hispanic Blacks had lower risk of being obese compared to US-born non-Hispanic Whites. Nevertheless, other studies have found similar results. For example, Vines and colleagues found that higher perceived racism was associated with a lower waist-to-hip ratio among African American women [39]. Another study found that greater discrimination was associated with poor physical functioning for White women, but not for Black Women [58]. Vines and colleagues also found a negative indirect effect of discrimination on physical health through self-esteem for White women only. Some researchers speculate that the awareness of discrimination may serve as a protective factor for non-Hispanic Blacks. Versey and Curtin [58] suggest that Whites are more likely to devalue the self as a consequence of unfair treatment, whereas Blacks may attribute experiences of unfair treatment to an unjust system rather than the self, which in turn can be protective to adverse health effects [58]. Contrary to Jackson et al.’s suggestion that Blacks may engage in unhealthy behaviors as stress coping strategies [59], it may also be the case that US born non-Hispanic Blacks may engage in healthy coping activities (e.g., exercise) that leads to some healthy outcomes, such as lower weight gain [39]. Further research is needed to examine the mechanism by which perceived discrimination may lead to lower obesity among US born non-Hispanic Blacks [53].

It is important to mention that US-born non-Hispanic Blacks still have higher rates of overweight/obesity compared to US-born non-Hispanic Whites [2]. There might be other psychosocial stressors that increase the risk of obesity for US-born non-Hispanic Blacks. US-born non-Hispanic Blacks report experiencing greater exposure to common stressors (e.g., financial strain, relationship problems) concurrent with greater exposure to race-related stressors (e.g., racial discrimination) than their White counterparts [60, 61]. A recent study shows that greater cumulative exposure to a wide range of stressors is associated with greater odds of obesity [35]. Future research needs to consider wider range of psychosocial stressors that may concurrently increase the risk of overweight/obesity for US-born racial/ethnic minority groups.

Compared to foreign-born non-Hispanic Whites, we found that foreign-born South Americans had higher risk of being overweight, whereas foreign-born Dominicans had lower risk of being obese at mean levels of perceived discrimination. Differences in migration patterns and sociopolitical histories, particularly among Hispanics/Latinos, may play a role in how foreign-born immigrants perceive and respond to unfair treatment attributed to race/ethnicity. The reasons for migration can affect how immigrants interact with their social environment [62]. Due to political and economic insecurity, large waves of Dominican immigrants have obtained permanent residency in the US [63, 64]. Dominican immigrants in the US suffer from high unemployment rates and other social barriers, such as poor access to care [65]. While racial discrimination may be a part of foreign-born Dominicans’ lived experiences, other stressors (e.g., employment stress) may play more prominent roles in their daily lives. Therefore, the health effects of discrimination may not be as potent compared to other groups. The inverse relationship between discrimination and obesity may also reflect unique coping behaviors to discrimination for Dominicans. However, very little research has examined coping behaviors within this Hispanic/Latino subgroup. Future research is needed to examine how Dominicans cope with race-related stressors like discrimination.

South Americans have differing migration patterns compared to Dominicans. They are more likely to enter the U.S. as professionals through employment-based visas [66]. Employment may expose South American immigrants to more instances of racial/ethnic discrimination in different social contexts (e.g., workplace, healthcare). This new stressor may influence foreign-born South Americans to engage in health-compromising coping behaviors. For instance, there is compelling evidence that poor sleep quality predict the development of obesity [67–69]. Limited research shows that ethnic discrimination is associated with daytime sleepiness, and short and long sleep duration among Hispanic/Latinos [70]. Individuals of South American background report shorter sleep durations and higher levels of sleepiness compared to other Hispanic/Latino subgroups [71]. It may be that exposure to discrimination may have a greater effect on obesity-related risk factors for South American immigrants. Nevertheless, future research is needed to better understand how nativity status may affect the relationship between perceived discrimination and obesity-related behaviors for this group.

There are limitations to consider in our study. We only had access to Wave 2 NESARC data and so this preliminary study was cross-sectional, which precludes the assumptions of causal associations between discrimination and obesity. It may be that those who are obese are more likely to experience racial/ethnic discrimination. However, Hunte found that everyday discrimination predicted an increase in waist circumference over time [36]. Our study examined only one aspect of discrimination. We used the Experiences of Discrimination scale, which captures discriminatory experiences that are acute and apparent [52]. It may be that acute forms of racial discrimination may have a greater influence overweight/obesity risk for certain groups, whereas chronic, more subtle forms of unfair treatment may be more deleterious to health for others [72]. Future studies should examine the effects that acute and chronic forms of discrimination may have on overweight/obesity across racial/ethnic groups. Discrimination may increase the risk of obesity through behavioral and psychological pathways [54]. The ways in which individuals respond to stressors may depend on their membership to racial/ethnic groups and nativity status. Future studies should examine the effects of discrimination on obesity-related behaviors (e.g., diet, sleep, physical activity) and how they may differ based on race/ethnicity and nativity. In addition, we only measured one aspect of acculturation (i.e., nativity). Acculturation is multidimensional construct and different acculturation process (e.g., degree to which one endorses the culture of the host country) may interact with social experiences and health differently [73]. Examining different aspects of acculturation (e.g., psychological acculturation, language use) would allow researchers to better understand immigrants’ experiences with discrimination.

Along with conceptual limitations, there are also methodological limitations to consider. The confidence intervals for some racial/ethnic groups, such as US-born Dominicans, were wide, which is indicative of the small sample sizes in the study. Our findings need to be replicated with larger sample sizes to better measure the association between discrimination and obesity. In addition, we did not adjust for urban/rural place of residence. Studies find the perception of discrimination and rates of obesity vary by urbanization [74–76]. Non-Whites are majorities in most urban counties [77], which may influence perception of discrimination for Whites and non-Whites. Future research should consider the level of urbanization in the relationship between perceived discrimination and obesity. Finally, this study used a single measure of adiposity (i.e., BMI) to assess obesity. While it is a reliable and valid measure that is commonly used to assess obesity-related morbidity and mortality [78–80], further research using additional adiposity measures would allow us to better capture obesity disparities and factors that help explain existing differences.

## Conclusion

Perceived discrimination may be a risk factor for obesity. Nativity status and race/ethnicity may play key roles in the relationship between perceived discrimination and overweight/obesity. Greater perceived discrimination was associated with lower risk of obesity among US-born non-Hispanic Blacks, whereas greater discrimination was associated with higher risk of overweight for foreign-born South Americans and lower risk of obesity among foreign-born Dominicans. More research is needed to identify the pathways that may link perceived discrimination to obesity among these groups. Perceived discrimination remains a significant stressor for racial/ethnic minorities and contributes to overall racial/ethnic health disparities. Psychosocial interventions aimed at reducing discrimination-related stress might help to reduce the obesogenic consequences of discrimination.

## Data Availability

De-identified data from Wave 2 interviews are openly available to researchers through National Epidemiologic Survey on Alcohol and Related Conditions after completing a data user agreement: https://www.niaaa.nih.gov/

## Abbreviations

BMI: Body mass index
CI: Confidence Intervals
EOD: Experiences of Discrimination
NESARC: National Epidemiologic Survey on Alcohol and Related Conditions
RRR: Relative risk ratio
SES: Socioeconomic status
US: United States
